# Recent Development in Production and Biotechnological Application of Microbial Enzymes

**DOI:** 10.1155/2015/280518

**Published:** 2015-03-02

**Authors:** Noomen Hmidet, Neelu Nawani, Sofiane Ghorbel

**Affiliations:** ^1^Enzyme Engineering and Microbiology Laboratory, National School of Engineering of Sfax, BP 1173, 3038 Sfax, Tunisia; ^2^Dr. D. Y. Patil Biotechnology and Bioinformatics Institute, Dr. D. Y. Patil Vidyapeeth, Pune 411033, India

Microbial enzymes are considered as a potential biocatalyst for several biotechnological applications. They are mainly characterized by their biochemical diversity and susceptibility to gene manipulation. Industries are often interested in new microbial strains producing different enzymes having original activities. The twelve articles published in this special issue balance the investigation of microbial enzymes having potential in several biotechnological and industrial applications.

By their study of the metagenomics of Indian buffalo rumen, K. M. Singh et al. showed the presence of a high potential source for biomass degradation enzymes. Plant cell wall degradation and biomass utilization provide genetic resource for degrading microbial enzymes that could be used in the production of biofuel. They identified potential contigs encoding biomass degrading enzymes including glycoside hydrolases, carbohydrate binding module, glycosyl transferase, carbohydrate esterases, and polysaccharide lyases.

The method of functional protein evaluation was used to identify enzymes for degrading each carbohydrate substrate. K. Yamamoto and Y. Tamaru identified several mannanases from* C. cellulovorans* for degrading LBG using functional protein evaluation and genomic data. In addition, they report that one of the identified mannanases, Man26E, contains a carbohydrate-binding module (CBM) family 59. As a result, four protein bands were classified into GH family 26 (GH26). One of the identified mannanases, Man26E, contains a carbohydrate-binding module (CBM) family 59, which binds to xylan, mannan, and Avicel.

Many papers in this special issue have focused on the optimization of the conditions for enzyme production and activity. In this section, H. Maalej et al. showed the production and biochemical characterization of a high maltotetraose (G4) producing amylase from crude enzyme preparation of* Pseudomonas stutzeri* AS22. The highest *α*-amylase production was achieved after 24 hours of incubation at 30°C. The formation of very high levels of maltotetraose from starch (98%) in the complete absence of glucose would have a potential application in the manufacturing of maltotetraose syrup.

In addition, S. L. T. Nguyen et al. reported the overexpression of a streptokinase from* Streptococcus pyogenes* DT7 in* Escherichia coli* and its highly effective renaturation and biochemical characterization. The streptokinase (SK) is emerging as an important thrombolytic therapy agent in the treatment of patients suffering from cardiovascular diseases.

Finally, N. Y. Stasyuk et al. reported a novel methylamine-selective amperometric bienzyme biosensor based on recombinant primary amine oxidase isolated from the recombinant yeast strain* Saccharomyces cerevisiae* and commercial horseradish peroxidase is described. The developed biosensor demonstrated good selectivity towards methylamine. The constructed amperometric biosensor was used for MA assay in real samples of fish products in comparison with chemical method.

Protease production and its biotechnological application represent one of the chapters of interest reported in this issue. Therefore, S. Ghorbel et al. characterized the crude extracellular proteases from the newly isolated* Streptomyces flavogriseus* HS1. HS1 strain produced at least five proteases. The crude extracellular proteases showed high stability when used as a detergent additive.

M. Jridi et al. in their paper reported the characterization and potential use of cuttlefish skin gelatin hydrolysates (CSGHs) prepared by different microbial proteases. Composition, functional properties, and* in vitro* antioxidant activities of these gelatin hydrolysates were investigated. The results reveal that CSGHs could be used as food additives possessing both antioxidant activity and functional properties.

R. Saxena and R. Singh in their paper reported the use of MALDI-TOF MS and CD spectral analysis for identification and structure prediction of a purified, novel, organic solvent stable, fibrinolytic metalloprotease from* Bacillus cereus* B80. The biochemical characterization of purified enzyme showed that the enzyme was able to hydrolyze various proteins with the highest affinity towards casein followed by BSA and gelatin. The enzyme exhibited strong fibrinolytic, collagenolytic, and gelatinolytic properties and stability in various organic solvents.

Laccases (*p*-diphenol-dioxygen oxidoreductases, EC 1.10.3.2) represent an interesting class of biocatalysts, being able to oxidize a wide spectrum of aromatic compounds along with reducing molecular oxygen to water. In their paper, C. Pezzella et al. immobilized crude laccase preparation from* Pleurotus ostreatus* on perlite and reported its efficient activity for Remazol Brilliant Blue R (RBBR) decolourisation in a fluidized bed recycle reactor.

G. Macellaro et al. reported the fungal laccases degradation of endocrine disrupting compounds. Over the past decades, water pollution by trace organic compounds has become one of the key environmental issues in developed countries. This is the case of the emerging contaminants called endocrine disrupting compounds (EDCs). EDCs are a new class of environmental pollutants able to mimic or antagonize the effects of endogenous hormones and are recently drawing scientific and public attention. In this study, five different EDCs were treated with four different fungal laccases, also in the presence of both synthetic and natural mediators. Mediators significantly increased the efficiency of the enzymatic treatment, promoting the degradation of substrates recalcitrant to laccase oxidation. Improvement of enzyme performances in nonylphenol degradation rate was achieved through immobilization on glass beads.

C. D. Anobom et al. reported in their review the importance of microbial lipases. These enzymes are highly appreciated as biocatalysts due to their peculiar characteristics such as the ability to utilize a wide range of substrates, high activity and stability in organic solvents, and regio- and/or enantioselectivity. These enzymes are currently being applied in a variety of biotechnological processes, including detergent preparation, cosmetics and paper production, food processing, biodiesel and biopolymer synthesis, and the biocatalytic resolution of pharmaceutical derivatives, esters, and amino acids. This review aims to compile recent advances in the biotechnological application of lipases focusing on various methods of enzyme improvement, such as protein engineering (directed evolution and rational design), as well as the use of structural data for rational modification of lipases in order to create higher active and selective biocatalysts.

B. Sharma et al. described the potential application of L-methionase against many types of cancers. This enzyme is an intracellular enzyme in bacterial species and an extracellular enzyme in fungi and is absent in mammals.

*Noomen Hmidet* *Neelu Nawani* *Sofiane Ghorbel*

